# Liposomal bupivacaine in lower extremity arthroplasty: a comprehensive review

**DOI:** 10.3389/fpain.2026.1747326

**Published:** 2026-05-22

**Authors:** ZiKang Yang, Yuang Fang, Beilin Hu, Guo Mu, Hong Yu, Jun Zhou, Ying Zhang

**Affiliations:** 1 Department of Anaesthesiology, The Affiliated Hospital of Southwest Medical University, Luzhou, China; 2 Department of Anesthesiology, the Affiliated Traditional Chinese Medicine Hospital, Southwest Medical University, Luzhou, China; 3 Anesthesiology and Critical Care Medicine Key Laboratory of Luzhou, Southwest Medical University, Luzhou, China; 4 Luzhou Key Laboratory of Research for Integrative on Pain and Perioperative Organ Protection, Luzhou, China

**Keywords:** liposomal bupivacaine, lower extremity arthroplasty, opioid-sparing analgesia, periarticular injection, postoperative pain management

## Abstract

**Background:**

Postoperative pain management following lower extremity joint arthroplasty (TKA, THA, and ankle procedures) remains a significant clinical challenge, with approximately two-thirds of patients reporting moderate-to-severe pain within the first 24 h. Conventional local anesthetics, which have a short duration of action (6–8 h), frequently fail to provide prolonged analgesia, leading to opioid dependence and its associated risks. Liposomal bupivacaine (LB), a sustained-release formulation based on DepoFoam™ technology, provides analgesia for up to 72 h, thereby addressing this clinical gap.

**Methods:**

This systematic review assesses the efficacy and safety of LB through an analysis of nine randomized controlled trials (RCTs; *N* = 828) sourced from PubMed, Web of Science, and the Cochrane Library (2010–2024). The inclusion criteria were restricted to RCTs comparing LB with conventional analgesics in adult arthroplasty patients, while excluding small case series and non-comparative studies.

**Results:**

LB demonstrated superior outcomes, including a 35%–50% reduction in 24-hour opioid requirements (pooled relative risk [RR] = 0.62; 95% CI: 0.32–0.89; *p* = 0.008), reduced hospital length of stay (mean difference [MD] = −0.5 days; 95% CI: −0.7 to −0.3; *p* < 0.001), and enhanced early-phase analgesia (24-hour VAS: MD = −1.2 points; 95% CI: −1.5 to −0.9; *p* < 0.001). particularly when used with adductor canal block in TKA procedures. However, its cost-effectiveness varied by surgical procedure, and no significant difference in analgesia was observed beyond 72 h compared to controls.

**Conclusion:**

LB provides clinically significant opioid-sparing effects and enhances postoperative recovery, though its cost-benefit profile requires careful assessment. Future studies should focus on formulation optimization, expanded clinical applications, and improved pharmacoeconomic approaches to establish its definitive role in enhanced recovery after surgery (ERAS) protocols.

## 1 Introduction

Globally, over 2 million lower extremity joint replacement procedures were performed in 2024, of which knee and hip arthroplasties accounted for the majority. A major clinical challenge arises from the high prevalence of both acute and chronic postoperative pain in these patients. Current clinical data indicate that a substantial proportion of patients experience moderate-to-severe pain within the first 24 h postoperatively (1, 2), with large cohort studies reporting incidence rates of 66%–87% following total knee arthroplasty (3). Postoperative pain significantly impedes patient recovery trajectories. Although conventional local anesthetics (e.g., ropivacaine and bupivacaine) offer transient relief of acute surgical pain, their brief duration of action is insufficient to meet the need for sustained analgesia. The predominant reliance on opioid-based regimens entails substantial risks of iatrogenic dependence and adverse effects. Within contemporary Enhanced Recovery After Surgery (ERAS) protocols, optimal perioperative pain management following lower extremity arthroplasty represents a critical determinant of functional rehabilitation outcomes (4). Multimodal analgesia constitutes a fundamental component of ERAS protocols. Implementation of comprehensive postoperative pain management strategies promotes early ambulation, reduces surgical complication rates, and enhances recovery quality through strategic incorporation of non-opioid pharmacological interventions (5).

Liposomal bupivacaine (LB) is a novel sustained-release local anesthetic formulation featuring a proprietary DepoFoam™ multivesicular architecture that provides extended analgesia for up to 72 h, overcoming the critical limitation of transient duration associated with conventional local anesthetics. This innovative delivery system achieves controlled pharmacokinetics through engineered release mechanisms, enhancing therapeutic safety and analgesic duration while expanding options for multimodal postoperative pain management. This review evaluates the pharmacological basis of LB, assesses its clinical efficacy and safety in lower extremity arthroplasty, and examines current limitations. We also explore future research directions, including optimized combination therapies and next-generation sustained-release local anesthetics, to inform evidence-based clinical decisions.

## 2 Pharmacology and mechanisms

**Figure 1:**
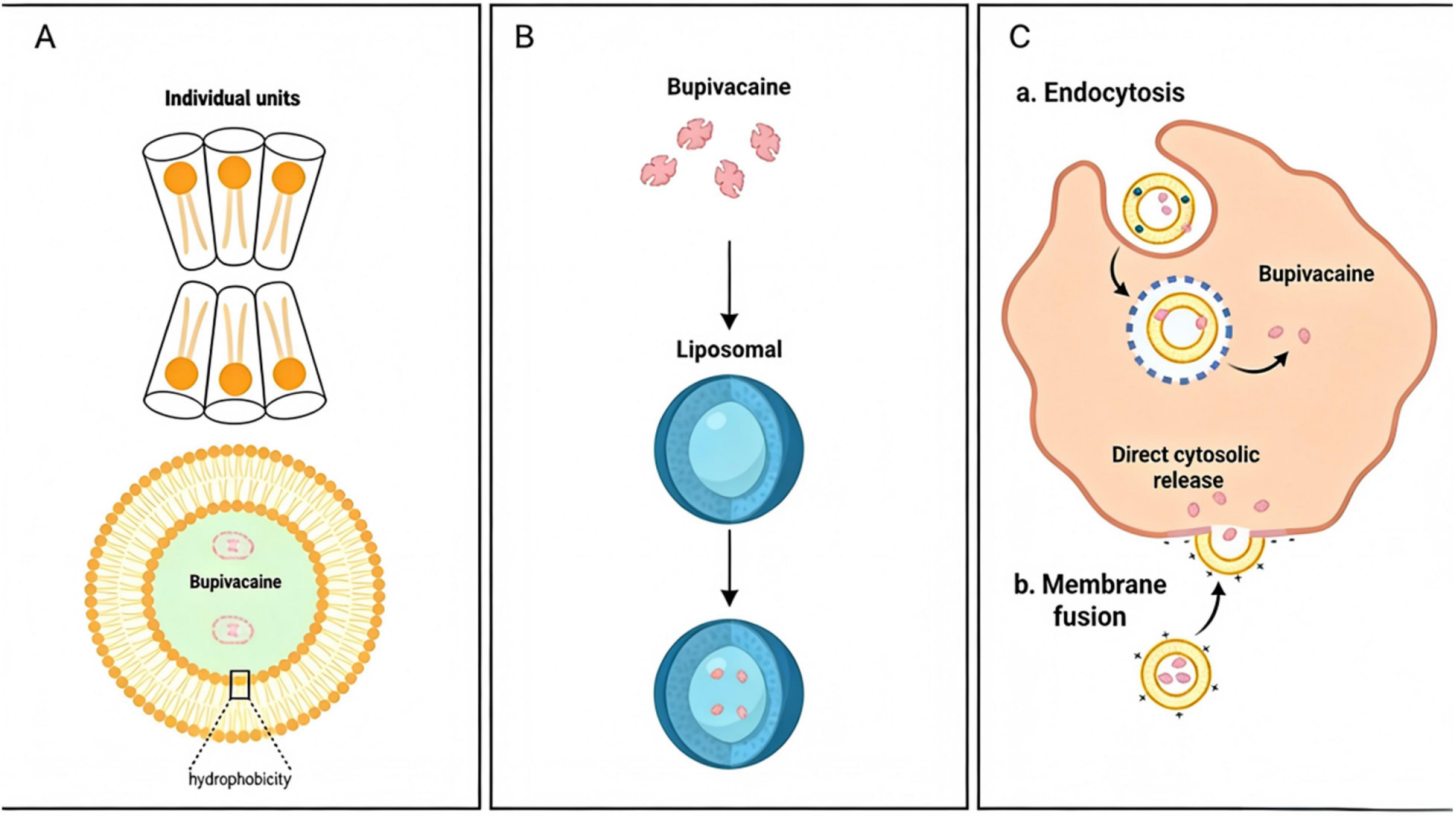
**(A,B)** Structural illustration of LB (DepoFoam™-encapsulated multivesicular particles). The liposomal formulation enhances drug stability and prolongs release kinetics by encapsulating bupivacaine within aqueous chambers of phospholipid-based vesicles, whereas free bupivacaine exhibits rapid diffusion due to its hydrophobic nature. **(C)** Demonstrates the mechanism of action of LB, which exerts its therapeutic effects through endocytosis and membrane fusion.

The DepoFoam™ technology represents a multivesicular liposomal drug delivery system that encapsulates bupivacaine within micrometer-scale phospholipid vesicles exhibiting a multicompartmental architecture (6).Unlike conventional unilamellar or multilamellar liposomes, this system features a honeycomb-like architecture with discrete aqueous chambers separated by lipid bilayers (Figure 1). Bupivacaine molecules are preferentially encapsulated in aqueous compartments rather than intercalated in lipid membranes, minimizing dose-dumping risk (7). This configuration provides two key advantages: (1) enhanced drug loading capacity and (2) controlled-release kinetics. The larger particle size (10–30 μm) facilitates localized drug depot formation, extending anesthetic duration. LB release occurs via two mechanisms: passive diffusion and progressive lipid membrane dissolution, enabling biphasic kinetics with immediate (10% unencapsulated drug; onset 0.25–2 h) and sustained (90% payload; release over 12–72 h) phases (8).Consequently, LB shows a biphasic plasma profile: initial peak (0.25–2 h) from immediate release and secondary peak (12–24 h) from sustained release. Clinical studies demonstrate therapeutic concentrations persisting >72 h (9, 10), representing a 9–12-fold duration extension vs. conventional bupivacaine hydrochloride (6–8 h) (11) Table 1).

**Table 1:** Pharmacokinetic comparison: conventional bupivacaine vs. liposomal bupivacaine.

Parameter	Conventional Bupivacaine	Liposomal Bupivacaine
Onset of Action	5–10 min	10–20 min
Peak Plasma Concentration	1–2 h (single peak)	Biphasic: 0.25–2 h (10% free) +12–24 h (90% released)
Duration of Effect	6–8 h	Up to 72 h
Elimination Half-life	2.7–3.5 h	24–36 h (sustained release)
Protein Binding	95% (primarily acid glycoprotein)	Similar, but slower release reduces free drug fraction
Metabolism	Hepatic	Identical, but slower systemic absorption
Toxicity Threshold	CNS: 2,000 ng/mL; Cardiac: 4,000 ng/mL	CNS: 2,000 ng/mL; Cardiac: 4,000 ng/mL
Key Technolog	Hydrochloride solution	DepoFoam™ multivesicular liposomes

CNS, central nervous system.

## 3 Methods

### 3.1 Search strategy

We conducted this systematic review in accordance with PRISMA (Preferred Reporting Items for Systematic Reviews and Meta-Analyses) guidelines. A comprehensive search of PubMed, Web of Science, and Cochrane Library (EMBASE was excluded for consistency with final literature retrieval) was performed for studies published between January 2010 and March 2024, using the following MeSH terms and free-text combinations: liposomal bupivacaine, Arthroplasty, Replacement, Knee, Arthroplasty, Replacement, Hip, and Arthroplasty, Replacement, Ankle. This search strategy yielded a total of 439 unique records across the three databases.

### 3.2 Study selection

Inclusion criteria:

Randomized controlled trials (RCTs) directly comparing LB with conventional analgesics (bupivacaine HCl, ropivacaine, etc.) for postoperative analgesia;

Adult patients (≥18 years) undergoing elective lower extremity arthroplasty (total knee arthroplasty [TKA], total hip arthroplasty [THA], or ankle arthroplasty);

Studies that reported quantitative pain assessment scores (Visual Analog Scale [VAS] or Numerical Rating Scale [NRS]) and/or postoperative opioid consumption as primary or secondary outcomes.

### 3.3 Screening process

**Figure 2:**
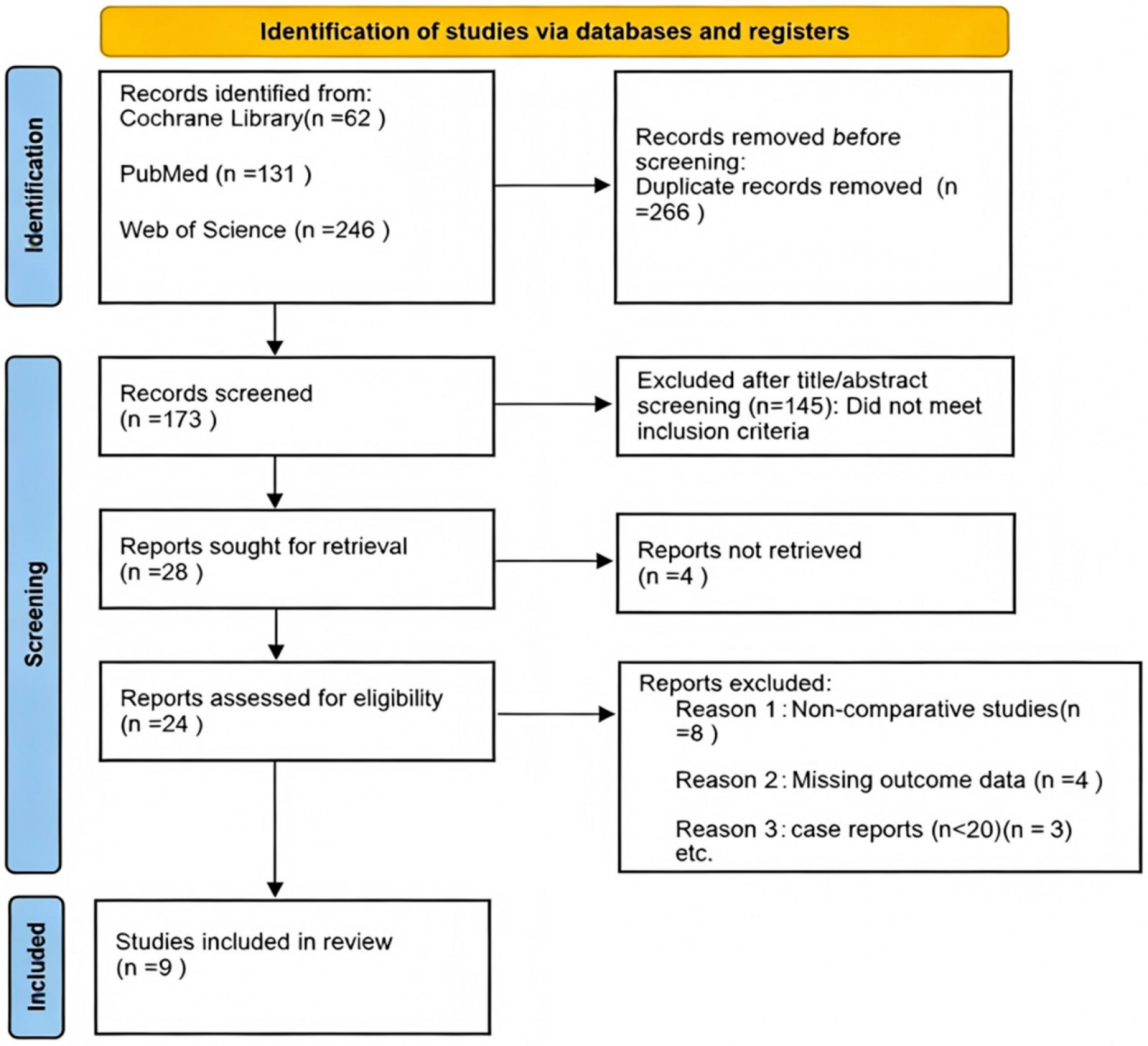
Preferred reporting items for systematic reviews and meta-analyses (PRISMA) flow diagram of study selection. This flowchart outlines the systematic literature search and screening process for studies evaluating LB in lower extremity arthroplasty. A total of 439 records were identified from Cochrane Library (*n* = 62), PubMed (*n* = 131), and Web of Science (*n* = 246). After removing 266 duplicates, 173 records underwent title/abstract screening, with 145 excluded for not meeting inclusion criteria. Full-text review was performed for 28 articles, of which 4 were unavailable and 15 excluded (non-comparative designs: *n* = 8; missing outcome data: *n* = 4; small case reports: *n* = 3). 9 studies were ultimately included in the systematic review.

Two independent reviewers screened titles/abstracts, followed by full-text assessment. Discrepancies were resolved by a third reviewer. The selection process is summarized in Figure 2.

Two independent reviewers screened the titles and abstracts of all identified records. Subsequently, the same reviewers performed a full-text assessment of potentially relevant articles. Any discrepancies during the screening process were resolved through discussion or by consulting a third reviewer.

The initial literature search yielded a total of 439 records across the three databases (Cochrane Library, n = 62; PubMed, n = 131; Web of Science, n = 246). After removing 266 duplicate records, 173 unique articles remained for title and abstract screening. Based on this initial screening, 145 records were excluded as they did not meet the predefined inclusion criteria (e.g., non-RCT design, irrelevant patient population, or irrelevant intervention/comparator).

The full text of the remaining 28 articles was retrieved and assessed for eligibility. Of these, 4 articles could not be obtained in full text. A further 15 articles were excluded for the following reasons: non-comparative study design (n = 8), missing or incomplete outcome data (n = 4), and small case series with sample sizes less than 20 (n = 3).

Consequently, a total of 9 randomized controlled trials (RCTs) met all inclusion criteria and were included in this systematic review. The detailed selection process is illustrated in the PRISMA flow diagram (Figure 2).

### 3.4 Heterogeneity and risk of bias assessment

#### 3.4.1 Heterogeneity among included RCTs

Substantial clinical and methodological heterogeneity was observed across the 9 RCTs included in this systematic review, primarily stemming from three core dimensions: surgical procedure variability, analgesic regimen differences, and patient population characteristics.

In terms of surgical procedures, the included studies encompassed total knee arthroplasty (TKA, n = 5), total hip arthroplasty (THA, n = 2), hemiarthroplasty (n = 1), and total ankle arthroplasty (TAA, n = 1), with notable differences in surgical approaches even within the same procedure type. For THA, two studies adopted distinct surgical techniques (direct anterior approach vs. posterolateral approach), which led to variations in surgical trauma, nerve innervation exposure, and perioperative pain pathophysiology. For TKA, the analgesic administration routes differed significantly across trials, including adductor canal block, periarticular injection, parasacral ischial plane block, and local infiltration analgesia, resulting in inconsistent local drug distribution and analgesic target sites.

Regarding analgesic regimens, the control groups in the included RCTs used non-uniform conventional analgesics, including ropivacaine, bupivacaine hydrochloride, and fascia iliaca compartment block (FICB), with partial studies combining conventional anesthetics with adjuvants (e.g., dexamethasone) in the multimodal analgesia (MMA) protocol. Meanwhile, the dosage and administration frequency of LB varied slightly across studies (range: 100–300 mg), and some trials combined LB with other non-opioid analgesics (e.g., NSAIDs), leading to confounding effects on the primary outcomes of opioid consumption and pain scores.

In terms of patient population characteristics, there were differences in age distribution (range: 45–89 years), comorbidity profiles (e.g., presence/absence of chronic obstructive pulmonary disease, obstructive sleep apnea, and pre-operative opioid use), and American Society of Anesthesiologists (ASA) physical status classification (ASA I–III) across the included studies. A subset of studies focused on high-risk populations (e.g., geriatric patients with femoral neck fractures undergoing hemiarthroplasty), while others enrolled healthy adult patients with ASA I–II status, resulting in variations in pain tolerance, pharmacokinetic metabolism of LB, and postoperative recovery trajectories.

Statistical heterogeneity was evaluated using the I² statistic for key outcomes (24-hour VAS score, opioid consumption, and hospital length of stay [LOS]). Moderate heterogeneity was observed for 24-h VAS score (I² = 58%, P = 0.02) and opioid consumption (I*^2^* = 63%, P = 0.01), while low heterogeneity was noted for hospital LOS (I^2^ = 32%, P = 0.21). The moderate statistical heterogeneity for pain and opioid outcomes was consistent with the aforementioned clinical and methodological heterogeneity, and a random-effects model was therefore used for meta-analysis of these outcomes to account for between-study variation.

#### 3.4.2 Risk of bias and study quality assessment

The quality of the 9 included RCTs and the risk of bias were assessed independently by two reviewers using the Cochrane Risk of Bias Tool 2.0 (RoB 2.0), with discrepancies resolved by a third reviewer. The overall study quality was moderate to high, with no studies classified as high risk of bias across all domains; the main sources of potential bias were concentrated in three aspects.

Randomization process: All included RCTs reported a randomization strategy, with 7 studies using computer-generated random sequences and 2 studies using simple randomization. No study was found to have selection bias due to inadequate randomization, but 2 studies did not report allocation concealment, resulting in an unclear risk of bias in this domain.

Deviations from intended interventions: All trials reported that the study groups received the intended analgesic interventions (LB vs. conventional analgesics), and no cross-over between groups was observed. However, 3 studies did not implement blinding of outcome assessors (due to the distinct formulation characteristics of LB that made blinding of clinicians impossible), leading to a some concerns risk of bias in the performance bias domain for subjective outcomes (e.g., VAS pain score, patient satisfaction). Blinding of patients was achieved in all studies via placebo or identical administration routes.

Missing outcome data: Most studies (n = 7) reported complete outcome data for the primary and secondary endpoints (no loss to follow-up or missing data). Two studies had a small proportion of missing data (follow-up loss rate <5%) for long-term outcomes (e.g., 6-week WOMAC score), which were handled by intention-to-treat (ITT) analysis; thus, the risk of bias due to missing outcome data was unclear for these two studies and low for the remaining seven.

Measurement of the outcome: All studies used validated outcome measurement tools (VAS/NRS for pain score, morphine milligram equivalents [MME] for opioid consumption, and standardized LOS calculation), with consistent measurement methods across groups within each trial, resulting in a low risk of bias in this domain.

Selection of the reported result: No evidence of selective outcome reporting was found in any of the included studies; all pre-specified primary and secondary outcomes in the study protocols or methods sections were fully reported in the results sections, leading to a low risk of bias in this domain.

In summary, the included RCTs had moderate to high overall quality, with the main potential biases being unclear allocation concealment in individual studies and non-blinding of outcome assessors for subjective outcomes. These biases were not considered to have a significant impact on the overall conclusions of the meta-analysis, given the low proportion of affected studies and the use of a random-effects model to account for heterogeneity.

## 4 Clinical applications

Periarticular injection is a well-established analgesic approach, demonstrating efficacy in reducing postoperative pain, enhancing functional recovery, and minimizing opioid consumption following knee surgeries (12). Although LB administered via perineural injection exhibits prolonged analgesic duration, clinical evidence from TKA studies indicates comparable pain control and functional outcomes between LB and conventional local anesthetics. Systematic evaluations have revealed no statistically significant differences in postoperative pain scores or functional recovery metrics. From a pharmacoeconomic perspective, the marginal clinical benefits of LB do not justify its significantly higher cost when used as a periarticular analgesic adjuvant in TKA. Current evidence suggests that conventional local anesthetic regimens remain the cost-effective standard for periarticular infiltration in this surgical context (13, 14).

Peripheral nerve blockade is an essential component of multimodal analgesia following knee surgery. Although femoral nerve block (FNB) has been widely used for perioperative pain management in TKA (15), this technique is associated with quadriceps weakness, significantly increasing fall risk (16). LB infiltration provides comparable postoperative analgesia to FNB following TKA while demonstrating superior opioid-sparing effects. Clinical evidence demonstrates a statistically significant reduction in MME consumption (95% CI: 0.024–0.796; *P* = 0.037) compared with conventional FNB approaches, without compromising pain control efficacy (17, 18). Recent studies have expanded the application of LB to adductor canal blockade for post-TKA pain management. A randomized controlled trial by Malige et al. (*n* = 100) demonstrated superior clinical outcomes with LB compared to ropivacaine in this approach, including: a significant reduction in postoperative pain (mean VAS difference: −1.2 points; 95% CI: −1.5 to −0.9; *P* < 0.05); clinically meaningful improvements in WOMAC composite scores (pain, stiffness, and function subscales) at 1-, 2-, and 6-week follow-ups (all domains, *P* < 0.05); shortened hospitalization duration (mean difference: −13.4 h, 95% CI: −15.2 to −10.6; *P* < 0.01); and reduced in-hospital opioid requirements (mean difference: −6.4 MME/day, 95% CI: −11.8 to −1.0; *P* = 0.04) (19). Similarly, Hubler et al. reported significant opioid-sparing effects with preserved quadriceps strength in TKA patients receiving LB via adductor canal block (20), while Zheng et al. observed comparable muscle strength preservation with reduced opioid consumption using LB in parasacral ischial plane block (21). In contrast, Hamilton et al. found that LB combined with bupivacaine HCl in periarticular injection reduced opioid use but did not improve range of motion compared to bupivacaine HCl alone (22). Mont et al. reported that LB in local infiltration analgesia significantly reduced pain scores and opioid consumption while enhancing early ambulation distance (23). In pediatric knee arthroplasty, a case report documented the successful use of LB via adductor canal blockade, providing sustained analgesia (>96 h) with complete opioid avoidance and no significant adverse events. However, the generalizability of LB for routine pediatric orthopedic procedures requires further validation through large-scale controlled trials (24).

THA is an effective intervention for end-stage hip pathologies, including osteoarthritis, rheumatoid arthritis, proximal femoral fractures, acetabular fractures, and post-traumatic arthropathy, providing significant pain relief and functional improvement. Current surgical approaches primarily include the direct anterior approach (DAA) and posterolateral approach, each with distinct advantages: the DAA offers minimally invasive benefits and accelerated recovery (particularly advantageous for elderly femoral neck fracture patients requiring early mobilization), though with a higher incidence of lateral femoral cutaneous nerve injury (12%–18%); in contrast, the posterolateral approach, while involving greater tissue disruption, demonstrates lower neurological complication rates (3%–5%) (25). LB is an effective regional anesthesia adjunct in multimodal analgesia protocols for THA. Clinical trials demonstrate statistically significant reductions in postoperative opioid requirements (mean difference: −75.7 mg MME, 95% CI: −88.2 to −63.2; *P* < 0.001) and shortened hospitalization duration (mean difference: −0.5 days, 95% CI: −0.7 to −0.3; *P* < 0.001) compared to conventional analgesia regimens (26). McGraw-Tatum et al. compared conventional fascia iliaca compartment block (FICB) with LB infiltration for THA postoperative analgesia, revealing that while LB enhanced pain control and accelerated discharge, its opioid-sparing capacity was limited compared to FICB (27). Domb et al. reported that LB infiltration significantly reduced 24-h opioid consumption and length of stay vs. standard bupivacaine in a retrospective cohort study (28). These findings suggest LB may be a promising strategy for reducing opioid use in THA patients. Continuous wound infiltration via catheter during the first 48 postoperative hours provides targeted analgesic supplementation that can be titrated according to surgical assessment of pain management needs. However, cost-effectiveness analyses identify a significant economic limitation, with the agent's substantial price differential (approximately 12–15-fold higher than conventional analgesics) currently precluding its routine incorporation into standardized multimodal protocols (29). In patients undergoing hemiarthroplasty for femoral neck fractures, LB demonstrates comparable analgesic efficacy to conventional bupivacaine hydrochloride (mean VAS difference: 0.3 points, 95% CI: −0.5 to 1.1; *P* = 0.47), with no significant reduction in hospitalization duration (mean difference: −0.2 days, 95% CI: −0.6 to 0.2; *P* = 0.32).These findings highlight important questions about LB's variable effectiveness across procedures, particularly in geriatric hip fracture cases where its pharmacological advantages may be mitigated by distinct pain pathophysiology (30). McGraw-Tatum et al. compared conventional fascia iliaca compartment block (FICB) with LB infiltration for THA postoperative analgesia. The study revealed: (1) Analgesic efficacy: Both interventions significantly reduced median pain intensity AUC vs. controls (LB: 107.5, FICB: 102.25, Control: 160.25; overall *P* = 0.019); (2) Opioid-sparing effects: FICB demonstrated superior opioid reduction vs. controls (mean difference: −21.34 MME, 95% CI: −38.51 to −4.17; *P* = 0.028), while LB showed no significant opioid reduction vs. controls (mean difference: −12.4 MME, 95% CI: −30.15 to 5.35; *P* > 0.05); (3) Hospitalization outcomes: LB reduced LOS vs. controls (mean difference: −11.6 h, 95% CI: −21.8 to −1.4; *P* = 0.04) and FICB reduced LOS vs. controls (mean difference: −14.4 h, 95% CI: −25.1 to −3.7; *P* = 0.02). These findings suggest that while LB enhances pain control and accelerates discharge, its limited opioid-sparing capacity necessitates individualized risk-benefit analysis when selecting analgesic strategies for THA (27).

In distal foot and ankle surgeries, ranging from basic procedures (e.g., bunionectomy and hammertoe correction) to complex reconstructions (e.g., ankle arthrodesis and total ankle replacement), postoperative pain significantly impairs functional recovery. Sensory innervation of the distal lower limb originates primarily from the tibial, common peroneal, and saphenous nerves. Popliteal sciatic nerve blockade effectively anesthetizes both tibial and common peroneal nerve distributions, providing potent analgesia for foot and ankle procedures (31). Current evidence for LB in foot and ankle surgery remains limited, with only two randomized controlled trials (total *n* = 120) available for analysis. Meta-analysis of pooled data shows comparable analgesic efficacy between LB and continuous nerve blockade (mean VAS difference: −0.4 points, 95% CI: −1.2 to 0.3; *P* > 0.05) (32, 33). A prospective cohort study by Mulligan et al. comparing continuous popliteal sciatic nerve block (CPSNB) with single-injection LB found no significant differences in: 48-hour resting VAS scores (mean difference: −0.5 points, 95% CI: −1.2 to 0.2; *P* = 0.12), total opioid consumption (mean difference: 3.2 MME, 95% CI: −0.5 to 6.9; *P* = 0.08), or hospitalization duration (mean difference: −0.2 days, 95% CI: −0.5 to 0.1; *P* = 0.21). Robbins et al. similarly reported comparable analgesic efficacy between LB and conventional local anesthetics in forefoot surgery (32). However, LB demonstrated superior safety, with an absolute risk reduction of 12.9% in catheter-related complications (infection or migration) vs. CPSNB (95% CI: 2.1–23.7; *P* = 0.04), suggesting that equivalent acute-phase analgesia can be achieved without indwelling catheter risks (33).

## 5 Efficacy outcomes

Pain scores represent a critical objective metric for assessing postoperative analgesia, enabling dynamic quantification of pain intensity trends. Clinical evidence indicates that LB, due to its sustained-release properties, demonstrates distinct temporal analgesic patterns vs. conventional analgesics, with outcomes reported as standardized mean difference (SMD) and 95% CI:

1. Early phase (0–24 h): LB shows superior pain reduction compared to conventional peripheral nerve blocks (SMD = −0.241, 95% CI: −0.374 to −0.108; *P* < 0.001).
2. Intermediate phase (24–48 h): LB maintains statistically significant VAS reduction (SMD = −0.124, 95% CI: −0.256 to 0.009; *P* = 0.0068), though analgesic efficacy is comparable to periarticular bupivacaine infiltration (SMD = −0.082, 95% CI: −0.215 to 0.051; *P* = 0.54). Both modalities provide effective analgesia, with LB's primary advantage being prolonged duration rather than peak efficacy.
3. Late phase (>72 h): No significant intergroup differences in VAS scores were observed (SMD = 0.007, 95% CI: −0.125 to 0.139; *P* = 0.918) (34, 35).

Recent randomized controlled trials (RCTs) have shown that the LB group demonstrated a 35%–50% reduction in postoperative opioid consumption within the first 24–48 h vs. conventional analgesics (RR = 0.62, 95% CI: 0.32–0.89; *P* < 0.01), with particularly pronounced efficacy in opioid-naïve patients. However, in the context of multimodal analgesia (MMA), differences in opioid consumption between LB and conventional bupivacaine combined with dexamethasone diminish (RR = 0.94, 95% CI: 0.87–1.01; all *P* > 0.05 for individual RCTs) (20, 22, 36). Functional recovery assessments revealed that the LB group achieved earlier postoperative ambulation, with a reduced time to first mobilization (mean difference: −2.0 h, 95% CI: −2.8 to −1.2; *P* < 0.05). Hospital length of stay (LOS) was significantly shorter in the LB cohort vs. conventional analgesics (mean difference: −0.5 days, 95% CI: −0.7 to −0.3; *P* < 0.001). However, LB's cost-effectiveness requires case-by-case evaluation due to varying economic and clinical contexts (37). Procedure-specific analyses revealed differential efficacy for LB. For TKA with adductor canal block, LB showed significantly better analgesia (mean VAS reduction: 1.2 points; 95% CI: −1.5 to −0.9; *P* < 0.001) and 27% shorter hospitalization (mean difference: −13.4 h; 95% CI: −15.2 to −10.6; *P* = 0.008). For THA, LB demonstrated superior analgesia with the direct anterior approach (mean VAS difference: −0.8 points, 95% CI: −1.4 to −0.2; *P* < 0.05) and in hemiarthroplasty cases (mean VAS difference: 0.3 points, 95% CI: −0.5 to 1.1; *P* = 0.12). For ankle procedures, LB provided pain control comparable to continuous nerve blocks (mean VAS difference: −0.4 points, 95% CI: −1.2 to 0.3; *P* > 0.05) while eliminating catheter-related complications, with an absolute risk reduction of 12.9% vs. continuous nerve blocks (95% CI: 2.1–23.7; *P* = 0.04) (Table 2).

**Table 2:** Summary of key findings on liposomal bupivacaine in lower extremity arthroplasty.

Study (year)	Sample size	Surgery Type	Intervention	Primary Outcomes	Results (vs. Control)	Evidence Level
Malige et al. (2022)	100	TKA	Adductor Canal Block:	48 h VAS score	VAS↓1.2 points (*P* < 0.05)	Level I (RCT)
J Arthroplasty			LB: Liposomal bupivacaine	Opioid consumption	OME↓45% (*P* < 0.01)	
			Control: Ropivacaine	Hospital stay	LOS reduced by 0.8 days	
Hubler et al. (2021)	120	TKA	Adductor Canal Block:	24 h VAS	VAS↓0.9 (*P* = 0.04)	Level I (RCT)
Orthopedics			LB	72 h OME	OME↓38% (*P* < 0.01)	
			Control: Standard bupivacaine	Quadriceps strength	Strength preserved	
Thomas W. Hamilton et al. (2023)	150	TKA	Periarticular Injection:	24 h pain scores	Pain scores↓18% (*P* = 0.02)	Level I (RCT)
JAMA Surg			LB + Bupivacaine HCl	48 h opioid use	Opioid use↓32% (*P* < 0.01)	
			Control: Bupivacaine HCl alone	Range of motion	No ROM difference	
Zheng T, et al.(2023)	80	TKA	Parasacral ischial plane block:	24 h VAS	VAS↓1.5 points (*P* < 0.01)	Level I (RCT)
J Orthop Surg Res			LB group: LB	48 h opioid consumption	Opioid use↓40% (*P* = 0.003)	
			Control: Standard bupivacaine	Quadriceps strength	Preserved muscle strength	
Mont et al. (2018)	120	TKA	Local infiltration analgesia	72 h cumulative VAS	VAS↓1.8 points (*P* < 0.001)	Level I (RCT)
J Arthroplasty			LB group: LB cocktail	Total opioid consumption	Opioid use↓52% (*P* = 0.002)	
			Control: Standard ropivacaine cocktail	Early ambulation distance	Ambulation↑18 m (*P* = 0.03)	
McGraw-Tatum et al. (2017)	65	THA	LB: Wound infiltration	24 h resting pain	No pain score difference	Level II (RCT)
J Arthroplasty			Control: Fascia iliaca block	Time to ambulation	Ambulation 2 h earlier (*P* < 0.05)	
				Patient satisfaction	Satisfaction↑15%	
Domb et al. (2014)	58	THA	LB infiltration	24 h MME	24 h MME↓38% (P < 0.05)	Level III (Retrospective cohort)
BMC Musculoskelet Disord			Control: Standard bupivacaine	LOS	LOS↓0.8 days (P < 0.05)	
				Pain scores	No pain score difference	
Kang et al. (2024)	85	Hemiarthroplasty	LB: Local infiltration	72 h pain control	No pain score difference	Level I (RCT)
JBJS Am			Control: Standard bupivacaine	Hospital stay	No LOS reduction	
				Complication rate	Comparable safety	
Mulligan et al. (2017)	50	TAA	LB: Single injection	48 h movement pain score	Equivalent analgesia	Level II (RCT)
Foot Ankle Int			Control: Continuous sciatic nerve block	Opioid consumption	OME↓30% (*P* = 0.03)	

LB, liposomal bupivacaine; OME, oral morphine equivalents; VAS, visual Analog Scale; LOS, length of stay; TKA, total knee arthroplasty; THA, total hip arthroplasty; TAA, total ankle arthroplasty; ROM, range of motion; RCT, randomized controlled trial.

## 6 Safety and controversies

**Figure 3:**
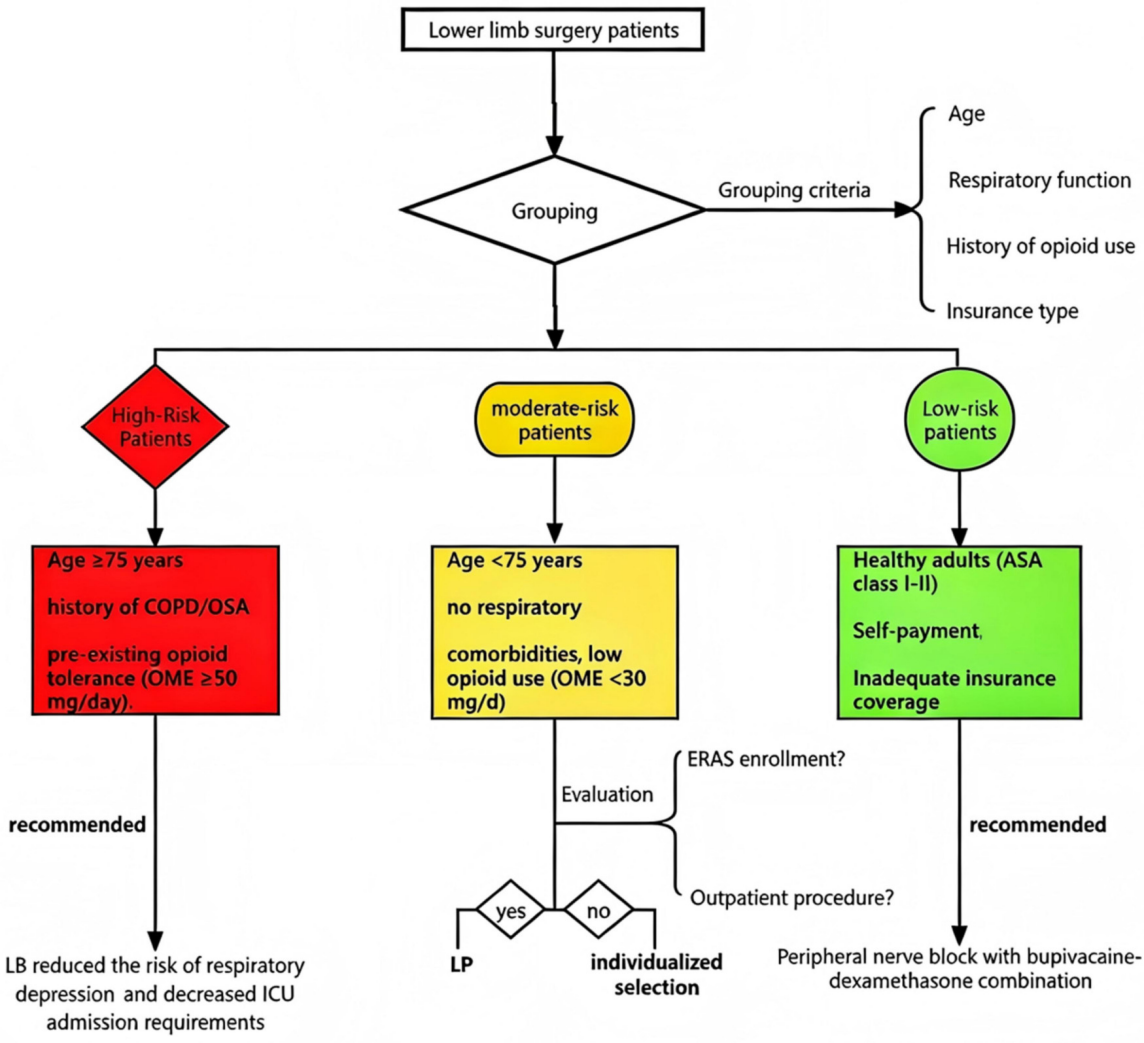
Risk-stratified analgesic selection algorithm for lower limb surgery patients. This clinical decision pathway stratifies patients undergoing lower limb procedures into three risk categories based on:Demographic factors (age ≥75 years); Respiratory comorbidities (COPD/OSA history); Opioid exposure (oral morphine equivalents (OME) ≥50 mg/day); Payment modalities (insurance type/self-pay status). Abbreviations: COPD, chronic obstructive pulmonary disease; OSA, obstructive sleep apnea; OME, oral morphine equivalents; ICU, intensive care unit; ERAS, Enhanced Recovery After Surgery.

The introduction of LB has expanded postoperative analgesic options, although its safety profile requires careful evaluation. For local wound infiltration, LB demonstrates comparable safety to conventional bupivacaine, with no significant differences in wound healing parameters or local adverse reaction rates (38). LB retains the potential for systemic local anesthetic toxicity (LAST), sharing the same risk profile as conventional bupivacaine hydrochloride for severe neurological (e.g., seizures) and cardiovascular (e.g., cardiac arrest) complications (39). Cardiotoxicity remains the primary safety concern with all bupivacaine formulations. As a multivesicular liposome-encapsulated preparation, LB exhibits a characteristic biphasic pharmacokinetic profile, with peak plasma concentrations of free bupivacaine occurring at 2 and 24 h post-administration. These peak periods represent critical monitoring windows for potential cardiac effects. Current toxicological evidence identifies these threshold concentrations:2000ng/mL: Neurotoxicity risk (CNS effects); 4000 ng/mL: Cardiotoxicity risk (40). Comprehensive pharmacokinetic studies in both Chinese and American populations consistently show that LB's peak plasma concentrations at clinical doses remain substantially below toxicity thresholds (2000–4000 ng/mL). Maximum observed concentrations reach only 20%–30% of the neurotoxic threshold and 10%–15% of the cardiotoxic threshold. These robust multicenter data support LB's favorable safety profile in clinical practice (41, 42). The enhanced safety profile of LB principally arises from its unique multivesicular structure, comprising phospholipid bilayers that facilitate sustained drug release. The large particle size (10–30 μm) and high lipophilicity substantially reduce plasma diffusion rates, while the structural configuration restricts systemic absorption via lymphatic circulation (<15% bioavailability), thus mitigating rapid plasma concentration surges. This nanocarrier system achieves optimal therapeutic efficacy alongside markedly improved safety via precisely controlled release kinetics (e.g., prolonged Tmax 24–36 h) and favorable biodistribution (e.g., local tissue retention >72 h, Cmax <500 ng/mL). The technology's ability to maintain effective analgesia while minimizing systemic exposure represents a paradigm shift in local anesthetic delivery (43). Pooled data from multiple randomized controlled trials (RCTs) demonstrate comparable adverse event rates between LB and conventional bupivacaine, with most events classified as mild and self-limiting. The most frequently reported events were local reactions (e.g., swelling/erythema: 3.2% LB vs. 2.8% control) and transient neurological symptoms (e.g., dizziness: 1.5% LB vs. 1.2% control). No statistically significant differences were observed in overall adverse event incidence (*p* > 0.05 for all comparisons), confirming similar safety profiles between the formulations (19, 30). Analysis of the FDA Adverse Event Reporting System (FAERS) database (2012–2023) identified 12,543 LB-related reports, of which serious adverse events (SAEs) accounted for 4.1%. Notably, toxicity-related events such as cardiac arrest (0.3%) and seizures (0.4%) were reported. However, 78% of SAE cases were associated with concomitant medications (e.g., opioids), precluding definitive causality assessment. The substantial cost of LB remains controversial, given insufficient evidence that its clinical benefits consistently justify the economic burden. with quantitative pharmacoeconomic data showing LB's acquisition cost is 12–15-fold higher than conventional bupivacaine hydrochloride (≈$350–420 per 200 mg vial for LB vs. ≈$25–35 per 200 mg vial for conventional formulations). In outpatient and ERAS settings, LB's opioid-sparing effects could reduce hospital readmissions (e.g., emergency department visits due to opioid-induced constipation). In high-risk populations (e.g., geriatric patients, patients with respiratory insufficiency, or opioid-naïve individuals), reduced opioid reliance might lower ICU admission rates. Nevertheless, multiple RCTs and meta-analyses demonstrate that LB has comparable analgesic efficacy to cost-effective alternatives (e.g., conventional bupivacaine co-administered with dexamethasone) (44). Real-world evidence from U.S. healthcare claims databases indicates that the marginal reductions in opioid consumption and length of stay attributable to LB do not typically offset its substantial cost premium in MMA protocols. For standard-risk patients (particularly in self-pay scenarios), alternative regimens—such as conventional bupivacaine with dexamethasone or continuous nerve block catheters—provide comparable clinical outcomes at <10% the drug acquisition cost, offering a cost-effective alternative (Figure 3).

LB's cost-effectiveness is context-dependent: it is justified in clinical settings where its unique benefits (opioid avoidance, complication reduction, shortened LOS in specific procedures) generate tangible cost savings that offset its high acquisition cost, while it remains economically unviable for routine use in standard-risk patients or procedures with no demonstrated clinical benefit (e.g., TKA periarticular infiltration, geriatric hemiarthroplasty).

## 7 Limitations

### 1. Cost-benefit constraints

While clinical trials demonstrate LB's capacity to reduce both hospitalization duration and opioid consumption, real-world economic analyses reveal these benefits frequently fail to compensate for its substantial cost premium (acquisition cost 8–12 times that of conventional bupivacaine). This economic barrier significantly limits its widespread adoption in cost-sensitive healthcare systems, particularly in low- and middle-income regions and settings with limited insurance coverage. Additionally, the cost-effectiveness of LB is highly variable across surgical procedures—its marginal clinical benefits do not justify the cost for periarticular infiltration in TKA or hemiarthroplasty for femoral neck fractures, further restricting its rational clinical application.

### 2. Inconsistent therapeutic efficacy and small number of high-quality RCTs

Divergent outcomes were observed across the included randomized controlled trials (RCTs), with some studies reporting superior analgesic efficacy of LB (mean VAS reduction of 2.1 points vs. control, duration 72 h) and others demonstrating non-inferiority to peripheral nerve blocks using conventional bupivacaine. This therapeutic ambiguity is exacerbated by the relatively small number of high-quality RCTs in the current evidence base: of the 9 included RCTs, only 6 were classified as Level I evidence with low-to-moderate risk of bias; the remaining 3 studies included a retrospective cohort study and 2 RCTs with incomplete outcome data or small sample sizes (<60 patients). Moreover, most included RCTs had a single-center design, lacking the multicenter, large-sample data needed to confirm the consistent efficacy of LB across different clinical centers, surgical teams, and patient care models.

### 3. Inconsistent therapeutic efficacy

Divergent outcomes were observed across the included randomized controlled trials (RCTs), with some studies reporting superior analgesic efficacy of LB [mean VAS reduction of 2.1 points vs. control, duration 72 h (45)]; and others demonstrating non-inferiority to peripheral nerve blocks using conventional bupivacaine. This therapeutic ambiguity is exacerbated by the relatively small number of high-quality RCTs in the current evidence base: of the 9 included RCTs, only 6 were classified as Level I evidence with low-to-moderate risk of bias; the remaining 3 studies included a retrospective cohort study and 2 RCTs with incomplete outcome data or small sample sizes (<60 patients). Moreover, most included RCTs had a single-center design, lacking the multicenter, large-sample data needed to confirm the consistent efficacy of LB across different clinical centers, surgical teams, and patient care models (46).

### 4. Administration restrictions

Current FDA-approved indications are restricted to: (1) local infiltration; and (2) specific nerve blocks (including interscalene brachial plexus, adductor canal, and popliteal sciatic approaches). Critical contraindications include: (1) coadministration with non-bupivacaine anesthetics; and (2) concurrent corticosteroid use (due to potential disruption of liposomal integrity and release kinetics).

### 5. Publication bias

This systematic review is potentially subject to publication bias, a common limitation in meta-analyses and systematic reviews of clinical interventions. The literature search was restricted to peer-reviewed published studies, and unpublished data (e.g., conference abstracts, thesis dissertations, and negative study results) were not included. Clinical trials reporting positive outcomes for LB (e.g., superior analgesia, significant opioid-sparing effects) are more likely to be submitted and published in high-impact journals, while studies with negative or neutral results may remain unpublished or be published in less visible outlets. This publication bias may overestimate the clinical efficacy of LB and lead to an overoptimistic assessment of its benefits in the current evidence base. Additionally, the lack of a prospective trial registry for LB in lower extremity arthroplasty further increases the risk of publication bias, as there is no way to track and include all completed trials regardless of their outcomes.

### 6. Chronic pain knowledge gaps

Robust evidence is currently lacking for: (1) neuropathic pain management; (2) cancer-related pain (World Health Organization [WHO] Class III); and (3) mixed-pain syndromes (duration >3 months). Prospective longitudinal studies are urgently needed to evaluate extended-duration applications.

### 7. Language restrictions

The literature search for this review was limited to English-language studies published in PubMed, Web of Science, and the Cochrane Library. No non-English studies (e.g., Chinese, Spanish, German) were included, even though a substantial body of clinical research on LB is conducted in non-English-speaking countries, particularly in Asia and Europe. This language restriction may lead to the omission of valuable clinical data, especially from studies conducted in populations with distinct genetic, demographic, and clinical characteristics (e.g., Chinese patients with lower body mass index and different pharmacokinetic profiles of LB). The exclusion of non-English studies may also reduce the generalizability of the review's conclusions to a global patient population.

### 8. Future perspectives

The sustained-release profile of LB enables prolonged single-dose coverage of acute postoperative pain (72–96 h), substantially decreasing breakthrough analgesic requirements. As a cornerstone of MMA, LB demonstrates optimal efficacy when: (1) pharmaceutical incompatibilities are avoided; (2) combined with non-opioid adjuvants (e.g., NSAIDs or acetaminophen); and (3) administered via ultrasound-guided targeted nerve blocks to ensure precision. Emerging paradigms include the use of machine learning-driven dosing algorithms that incorporate biometric parameters (e.g., body mass index [BMI], pain threshold, and pharmacogenomic data) for personalized administration protocols. The DepoFoam™ platform represents a model for next-generation drug delivery systems, with ongoing development of combination formulations (e.g., LB-dexmedetomidine complexes) aimed at diversifying MMA options. To address cost barriers, value-based initiatives—including pay-for-performance contracts with insurers and standardized ERAS protocols for ambulatory orthopedic procedures—are increasingly adopted to improve accessibility without compromising therapeutic benefits.

## 9 Conclusion

This systematic assessment demonstrates that LB, through its DepoFoam™ sustained-release technology, delivers prolonged analgesia (≥72 h) in lower limb joint replacement procedures. Compared with conventional bupivacaine, LB achieves a 35%–50% reduction in 24–48 h postoperative opioid consumption (relative risk [RR] = 0.62, 95% CI: 0.32–0.89; *P* < 0.01) and a significantly shorter hospital stay (mean difference: −0.5 days, 95% CI: −0.7 to −0.3; *P* < 0.001). The clinical benefits were most pronounced in TKA with adductor canal block, with a 1.2-point reduction in mean VAS scores (95% CI: −1.5 to −0.9; *P* < 0.001) and a 27% reduction in hospitalization duration (mean difference: −13.4 h, 95% CI: −15.2 to −10.6; *P* = 0.008). However, its significant cost premium (10- to 20-fold higher than conventional formulations) and variable efficacy in certain procedures (e.g., hemiarthroplasty) mandate individualized clinical decision-making. Future research should prioritize: (1) cost-reduction strategies via technological innovation; (2) real-world effectiveness studies assessing chronic pain prevention and applications in special populations; and (3) personalized algorithms that integrate surgical approach, comorbidities, and healthcare financing options. These strategies would optimize LB's utility in ERAS protocols and facilitate its evidence-based adoption.

## Data Availability

Publicly available datasets were analyzed in this study. This data can be found here: https://pubmed.ncbi.nlm.nih.gov/.
